# Maltose-Binding Protein Fusion Allows for High Level Bacterial Expression and Purification of Bioactive Mammalian Cytokine Derivatives

**DOI:** 10.1371/journal.pone.0106724

**Published:** 2014-09-08

**Authors:** Andrea Pennati, Jiusheng Deng, Jacques Galipeau

**Affiliations:** 1 Department of Hematology and Medical Oncology, Emory University, Winship Cancer Institute, Atlanta, Georgia, United States of America; 2 Department of Pediatrics, Emory University, Winship Cancer Institute, Atlanta, Georgia, United States of America; Instituto Butantan, Brazil

## Abstract

Fusokines are chimeric proteins generated by the physical coupling of cytokines in a single polypeptide, resulting in proteins with highly pleiotropic activity and the potential to treat cancer and autoimmune ailments. For instance, the fusokine GIFT15 (GM-CSF and Interleukin 15 Fusion Transgene) has been shown to be a powerful immunosuppressive protein able to convert naïve B cells into IL-10-producing B cells. To date, the mammalian cell systems used for the expression of GIFT15 allow for secretion of the protein in the culturing media, an inefficient system for producing GMP-compliant fusokines. In this study we report the bacterial expression of bioactive recombinant GIFT15 (rGIFT15). Indeed, there is a constant demand to improve the expression systems for therapeutic proteins. Expression of a maltose-binding protein (MBP) fusion protein efficiently allowed the accumulation of soluble protein in the intracellular milieu. Optimizing the bacterial culture significantly increased the yield of recombinant protein. The biological activity of rGIFT15 was comparable to that of fusokine derived from a mammalian source. This approach led to the production of soluble, endotoxin-free functional protein, averaging 5 mg of rGIFT15 per liter of culture. This process is amenable to scale up for the development of Food and Drug Administration (FDA)-compliant immune-modulatory rGIFT15.

## Introduction

Recombinant human cytokines such as G-CSF and IL-2 are routinely used as pharmaceuticals to restore or augment the immune system and combination cytokine therapies have been tested as cancer treatments [1], [2]. Over the past decade, we have developed a novel engineered biopharmaceutical platform – fusokines – for cell-base immune therapy. Fusokines are the fusion of two distinct cytokines in a single polypeptide that results in synergistic bioactivity not observed by the use of parent cytokines [3]–[7]. The therapeutic goal of this new class of protein pharmaceuticals is to treat ailments with unmet medical needs, i.e. cancer and autoimmune disease [3], [5]–[17].

We have shown that the coupling of GM-CSF and the N-terminal truncated version of MCP-1/CCL2, aka GMME1, deploys potent immunosuppressive properties. In vivo the protein was able to reverse proinflammatory factors identified in a mouse model of rheumatoid arthritis [13], [14]. Alternatively, the fusion of GM-CSF with CC-chemokine MCP3 (hereafter GMME3) generates a protein that allows for conversion of naïve B-cells to a novel suppressor phenotype [8].

Likewise, when IL-2 is fused to the ectodomain of TGFβ receptor II (FIST fusokine, in this instance FIST-2) the chimera is a bioactive protein that teams anti-angiogenesis to an immune antitumor response by activating IL-2-receptor expressing cells [11], [12].

The fusion of GM-CSF with the common γ chain interleukins (IL-2, IL-9, IL-15 and IL-21) as a chimeric protein leads to gain-of-function properties when compared to the bioactivity of GM-CSF and the interleukins individually. These GIFTs (GM-CSF and Interleukin Fusion Transgenes) typically lead to STAT hyperphosphorylation in responding lymphomyeloid cells [3], [7], [16]–[18]. The best-characterized member of this family is GIFT15, a fusion of GM-CSF and IL-15 [15], [16], [18].

Mammalian cell lines genetically engineered to express GIFT15 produce a secreted glycoprotein of 282 amino acid residues and molecular weight of 55 kDa [18]. Biochemically, GIFT15 binds atypically to the trimeric IL-15 receptor, a process probably caused by physical interference of the GM-CSF moiety at the N-terminus of the chimera. As a result, asymmetrical signaling takes place downstream of the IL-15 receptor, leading to the hyperactivation of STAT3 and suppression of common γ chain-mediated STAT5 phosphorylation [18]. This co-opting of IL-15R signaling machinery leads to an unusual cell physiological response, chiefly the ability of GIFT15 to reprogram naïve B cells into regulatory CD1d^+^/CD5^+^ B cells that secrete IL-10 [15], [16], [18], [19]. The possibility of generating *ex vivo* GIFT15-derived regulatory B cells with immunosuppressive properties is an attractive prospective autologous cell therapy for autoimmune diseases such as multiple sclerosis [20], [21].

Unfortunately for many pre-clinical protein pharmaceutical candidates with high therapeutic potential, few will ever be tested in clinic [22]. One of the major obstacles to clinical development is the excessive cost to produce GMP-grade protein pharmaceuticals. As a result, there is an urgent need to apply new or proven methodologies, to facilitate the first-in-human assessment of potential therapeutics in a rapid and cost efficient way. Akin to other FDA-approved recombinant cytokines available on the market, GIFT proteins can be manufactured utilizing eukaryotic cell expression systems with downstream processing and protein purification [18]. However, the use of transfected mammalian cells and costly downstream processing to achieve high purity proteins leads to disadvantageously high GMP-compliance costs that impede the industrial development of these agents.

Bacterial expression systems are suitable for cost effective and safe production of recombinant proteins on a large scale, providing unique advantages for pharmaceutical proteins that are required in large amounts and whose production is too expensive for conventional manufacturing processes [23], [24]. Different species and strains of bacteria have been used to produce many valuable recombinant proteins, including subunit vaccines, antibodies and antibody fragments, hormones, blood products, cytokines and enzymes [25]. Recombinant proteins produced in *E. coli* nonetheless lack certain post-translational modifications which are present in many eukaryotic proteins. Glycosylation is the most common post-translational modification thus affected and GIFT15 is highly glycosylated [16]. Although the absence of bacterial N- or O-glycans were initially thought to limit the therapeutic potential of bacteria-derived leukines, several cytokine products have now been tested in the clinic and in certain cases the absence of glycans does not affect the biological activity of the recombinant protein [25]. In this report, we developed a method to generate and purify bacterial-derived GIFT15 (rGIFT15) and further demonstrate that it maintains potent bioactivity despite the absence of any eukaryotic post-translational modification. Our method of manufacture and purification clearly demonstrates that rGIFT15 exploits pharmaceutical properties akin to those of eukaryotic-sourced recombinant protein and allows for high scale, cost-effective and GMP-compliant production of cytokine derivatives.

## Materials and Methods

### Ethics Statement

All animal experiment were approved by the Emory Institutional Animal Care and Use Committee and performed by accepted veterinary standards. C57BL/6 mice were sacrificed using CO_2_ and splenocytes were harvested from spleen.

### Bacterial strains, plasmids and animals

*E. coli* Rosetta(DE3)pLysS, BL21(DE3)pLysS, Origami(DE3)pLysS and Rosetta-gami 2(DE3)pLysS bacterial strains were from Novagen. The pET vector series (pET16b, pET20b, pET32b and pET42b) was purchased from Novagen and pMALc4x was from New England Biolabs (UK). All experimental C57BL/6J mice were purchased aged 6 to 8 weeks (The Jackson Laboratory, Bar Harbor, Maine). All chemicals were of reagent grade and were obtained from commercial sources. Mouse IL-10 ELISA Ready-SET-Go! was purchased from EBioscience.

### Construction of vectors for expression of GIFT15

The open reading frame of GIFT15, devoid of the GM-CSF signal peptide, was amplified by PCR from a retroviral vector containing his cDNA using Phusion Hot Start Flex DNA Polymerase (NE BioLabs) and subcloned in different expression vectors. Vector-compatible primers harboring specific restriction enzyme recognition sites (*Nde*I-5′and *Xho*I-3′ for pET20b/pET16b, *Bam*HI-5′ and *Xho*I-3′ for pET32a/pET49b and *Eco*RI-5′ and *Pst*I-3′ for pMalC4x) were designed and commercially synthesized (Integrated DNA Technologies, Coralville, IA). Additional primers were designed to add a His-tag in front of the maltose binding protein and replace the Factor Xa recognition site by the one recognized by the TEV protease in pMALc4x (Tobacco Etch Virus protease produced as previously described [26]). Purified PCR fragments (QIAquick PCR Purification Kit) were treated with 1.5 units of appropriate restriction enzymes (NE BioLabs). Vectors were also digested with appropriate restriction enzyme for 2 h at 37°C. Following gel purification, the linearized plasmids were dephosphorylated with 1 unit of Antarctic Phosphatase (NE BioLabs) at 37°C for 1 h. The linearized vectors were then mixed with the fusokine gene (molar ratio 1∶3), 1 unit T4 DNA Ligase (NE BioLabs), and incubated at 16°C overnight. The ligation reaction was then used to transform *E. coli* DH5α competent cells (NEB) and positive clones were selected on Luria-Bertani (LB) agar plates supplemented with the appropriate antibiotic. After confirming the integrity of the fusokine constructs by sequencing, expression vectors were introduced into different *E. coli* strains for protein production.

### Protein expression trials

Expression of recombinant fusokine was optimized by varying bacterial growth and induction conditions. Bacterial colonies, transformed with the different expression vectors, were picked and used to inoculate 1 mL LB medium which was cultured overnight in appropriate antibiotic at 37°C. The following morning, 1 mL of each overnight culture was transferred to 50 mL of either LB, Minimal (M9) or Terrific Broth (TB) medium with antibiotics. After reaching OD_600_ = 1, the cultures were cooled down to 25 or 18°C or alternatively maintained at 37°C and IPTG was added (concentration between 0.05 to 1 mM). The cultures were grown for an additional 3 to 16 h at 220 rpm. The cells were then centrifuged and the pellet was re-suspended in 5 mL of BugBuster Master Mix (EMD Millipore). After incubation on ice for 15 min, the crude extract was clarified by centrifugation. These crude bacterial extracts were analyzed by polyacrylamide gel electrophoresis (SDS–PAGE) under denaturing conditions and Western blotting (see below).

### Expression and Purification of rGIFT15

*E. coli* Rosetta-gami 2(DE3)pLysS cells, harboring the plasmid pHisMBP-GIFT15, were used to inoculate 50 mL of TB medium containing ampicillin (100 mg/L) and chloramphenicol (34 mg/L), at 37°C overnight. The next day, 5 mL of the starter culture was used to inoculate 4×0.5 L of the same liquid culture medium at 37°C, 220 rpm. After the culture reached an OD_600_ between 0.8 and 1 (typically 5 h), the culture temperature was lowered to 25°C and IPTG was added to a final concentration of 0.2 mM. Expression of rGIFT15 was induced for 16 h. Cells were harvested by centrifugation at 40,000 g in a Sorval SS34 rotor for 20 min at 4°C. Typically, 15–20 g of wet cell paste was obtained from 2.0 L of bacterial culture. Unless otherwise stated, all protein purification steps were carried out at 4°C. Cell lysate was prepared by resuspending cell pellets in approximately 15 volumes of lysis buffer (20 mM sodium phosphate pH 7.4, 20 mM imidazole, 100 mM NaCl, 1 mM PMSF, 1 mM β-mercaptoethanol and 1X BugBuster Protein Extraction Reagent - EMD Millipore) and sonicating on ice for 5 min (2 min on and 1 min off cycles). Crude extract was clarified by centrifugation at 30,000 g for one hour at 4°C. All chromatography experiments were performed at 4°C with an AKTA purifier (GE Healthcare). The cell-free extract, typically 60–80 mL, was adjusted to pH 7.4 and applied slowly onto a pre-equilibrated 5 mL HisTrap chelating HP column (GE Healthcare) charged with Ni^+2^ (buffer A, 20 mM sodium phosphate pH 7.4, 10 mM imidazole, 500 mM NaCl and 1 mM β-mercaptoethanol). The column was then washed with 10 column volumes (CV) of buffer. A step gradient of 5% buffer B (20 mM sodium phosphate pH 7.4, 500 mM NaCl, 500 mM imidazole and 1 mM β-mercaptoethanol) removed further contaminant proteins. The fusokine was eluted with 150 mM of imidazole. The purified protein sample was cleaved overnight at 4°C with TEV protease. Following the cleavage reaction, the mixture containing the target protein was concentrated and then diluted with 20 mM Tris-HCl pH 8.0, then applied to 10 mL DEAE Sepharose in FPLC equilibrated in 20 mM Tris-HCl, pH 8.0. The cleaved protein eluted at approximately 20% buffer B (20 mM Tris-HCl pH 8.0, 1 M NaCl). The purification process described above typically yielded high purity (>95%) protein samples. To remove endotoxin, the protein solutions were treated with 1% Triton X-114 on ice. After warming the reaction to 37°C, the detergent phase containing the endotoxins was precipitated by centrifugation at 3,000 g for 15 min, and the fusokine was recovered in the aqueous phase. Alternatively, the column was washed with 40 column volumes of the same binding buffer (20 mM sodium phosphate pH 7.4, 10 mM imidazole, 500 mM NaCl and 1 mM β-mercaptoethanol) but 0.1% triton X-114 was added to the buffer. Elution was achieved with the same 150 mM imidazole [27]. The degree of endotoxin contamination was determined using an LAL Chromogenic Endotoxin Quantitation Kit (Pierce). The purified protein solution was then desalted on a PD-10 column (GE Healthcare) equilibrated in sterile PBS and the sample was filter sterilized prior to use.

### Western blot and ELISA

For cytokine stimulation assays with murine B lymphocytes, cells were seeded at a density of 2.5×10^6^/mL in 96-well plates, with a well solution volume of 200 µl. Cells were starved in serum-free medium for 2 hours, then were stimulated with either 0.1 nM of GM-CSF, 0.1 nM of IL-15, or both cytokines, or 0.1 nM of GIFT15 for 1 h at 37°C. Cells were collected, washed once with ice-cold PBS, and lysed in buffer supplemented with protease inhibitors and phosphatase inhibitor. Cell lysates were separated by SDS-PAGE, and Western blot analysis was performed with α-phosphorylated STAT3 and STAT5 antibodies (Cell Signaling, Danvers, MA). STAT3 antibody (Cell Signaling) detection was used as a loading control. Blotted proteins were visualized using enhanced chemiluminescence (ECL; Amersham, Amersham, UK). For detection of multiple STAT family members, the membranes were stripped of bound antibody and reprobed with antisera. For the quantification and identity of the recombinant GIFT15 protein, mouse IL-15 and GM-CSF antibodies were used (R&D Systems, Minneapolis, MN) and recombinant mouse IL-15 and/or mouse GM-CSF proteins were used as standards (R&D Systems, Minneapolis, MN).

### Conversion of naïve splenic B cells to regulatory cells

Splenocytes were collected from normal C57Bl/6 mice and B cells were isolated following the EasySep Mouse B Cell Isolation Kit protocols (STEMCELL Technologies Inc., Canada). B cells were cultured in RPMI 1640 medium (Thermo Scientific, Waitham, MA) supplemented with 10% fetal bovine serum (Wisent Bioproducts, St. Bruno, Canada), 1% penicillin-streptomycin (Thermo Scientific), 1 mM sodium pyruvate, non essential amino acids, 20 mM Hepes and β-mercaptoethanol (Thermo Scientific) as well as rGIFT15 in a 5% CO_2_ incubator for 4–5 days. The phenotype of the B cells was analyzed by using a BD FACSCanto II flow cytometer (San Jose, CA) following incubation with the appropriate antibodies for 20 min at 4°C.

## Results

### Expression of recombinant mouse GIFT15

**Figure 1:**
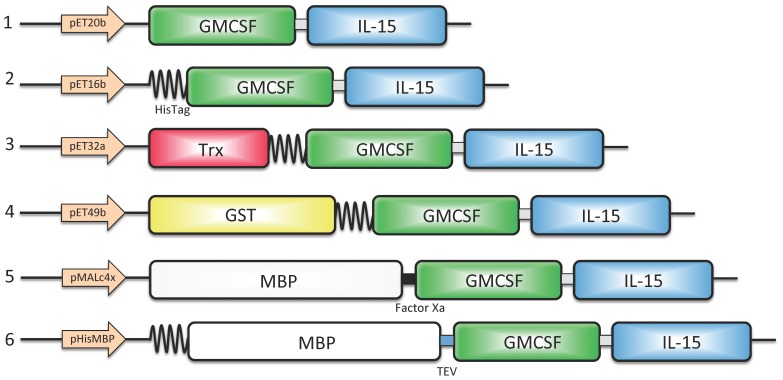
Schematic representation of the plasmid vector constructs tested for expression of mouse GIFT15 in *E. coli*. 1. pET20b: expression of GIFT15 without any tag, GM-CSF domain (lacking the signal peptide, green box) and IL-15 domain (blue box). The linker between the two domains is formed from the signal peptide and propeptide of IL-15 (grey box). 2. pET16b: expression of GIFT15 with an additional His-tag (6x-His) at the N-terminus of the fusokine. 3. pET32b: expression of GIFT15 downstream of thioredoxin (TRX, red box) followed by a His-tag. 4. pET42b: expression of GIFT15 in fusion with glutathione S-transferase (GST, yellow box). 5. pMALc4x: expression of GIFT15 in fusion with the maltose-binding protein (MBP, white box). 6. pHisMBP: expression of GIFT15 with the addition of a His-tag at the N-terminus of the MBP domain and replacement of the Factor Xa protease recognition site with that of the TEV protease.

The expression of a cDNA encoding mouse GIFT15 was initially tested using the pET20b (Novagen) expression vector under the control of the T7 lac promoter. A translational start codon (ATG) was inserted after the GM-CSF signal peptide (Fig. 1). Using this methodology, attempts to isolate the fusokine were unsuccessful. Western blot analysis showed that the protein did not accumulate significantly after induction with IPTG. In order to improve protein yield, the gene encoding GIFT15 was subcloned into pET16b (Novagen) vector that allows for expression of the protein as an N-terminal extension of a His-tag. The small His-tag also did not prevent the degradation of GIFT15 in the intracellular environment, and only traces of recombinant GIFT15 could be detected by immunoblot analysis. The lability of GIFT15 prompted us to radically modify the expression system in order to increase the amount of soluble protein and improve its stability in the bacterial intracellular milieu. Gene fusion is an approach that has been successfully used for producing soluble heterologous proteins in *E. coli* [28], therefore the GIFT15 gene was subcloned in three different vectors that combine the expression of GIFT15 with thioredoxin (TRX), maltose-binding protein (MBP) or glutathione S-transferase (GST) (Fig. 1). Expression of these GIFT15 fusion proteins was carried out using different *E. coli* strains, growth and induction conditions, and the expression levels were evaluated by SDS-PAGE and Western blot analysis. Only the MBP fusion construct resulted in the successful overexpression of GIFT15 as soluble protein in the intracellular environment. Moreover, the amount of soluble recombinant protein increased greatly by lowering the induction temperature to 25°C. The highest level of expression was induced with 200 µM IPTG and it was significantly affected by the host *E. coli* strain. Optimal results were obtained using Rosetta-gami (DE3)pLysS (Novagen) cells. The effect of culture medium composition on the production of the fusokine GIFT15 was investigated. Cells grown in LB, TB or M9 media at 25°C were analyzed. The largest amount of MBP-GIFT15 was obtained growing cells in TB medium.

**Figure 2:**
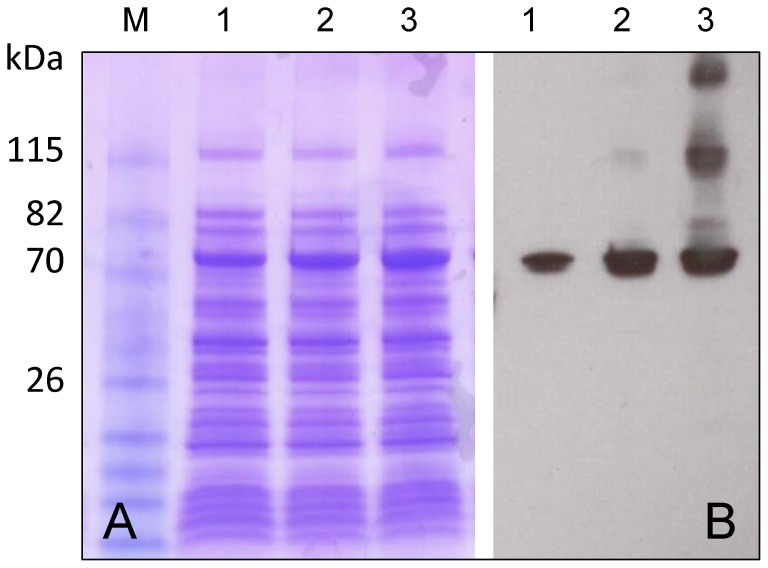
Heterologous expression of His-MBP-GIFT15 in Rosetta-gami (DE3)pLysS. (A) SDS-PAGE and (B) Western blot analysis. Soluble fractions after (1) 1 h of induction with IPTG; (2) after 3 h of induction; (3) after 16 h of induction. Immunodetection was conducted with anti-mouse IL-15 antibody.

Although the MBP-tag forced the accumulation of soluble fusokine, this tag proved inefficient for protein purification. Additionally, non-specific cleavage of the fusion protein by the Factor Xa protease was observed. To increase the recovery of soluble MBP-GIFT15, a polyhistidine-tag was added to the N-terminal region of the MBP carrier [29]. Furthermore, the Factor Xa protease recognition site was replaced with that of the TEV protease. The addition of the His-Tag to the N-terminal of MBP and/or the replacement of the protease cleavage site did not influence the expression level of soluble recombinant MBP-GIFT15 (Fig. 2) and was permissive for efficient purification.

SDS-PAGE analysis revealed the accumulation of ∼73-kDa protein after 1 hr of induction. The intensity of the induced bands increased with time of induction and western blot analysis confirmed that this band was the fusion MBP-GIFT15 protein with the expected molecular weight (73 kDa).

### Purification of recombinant mouse GIFT15

**Figure 3:**
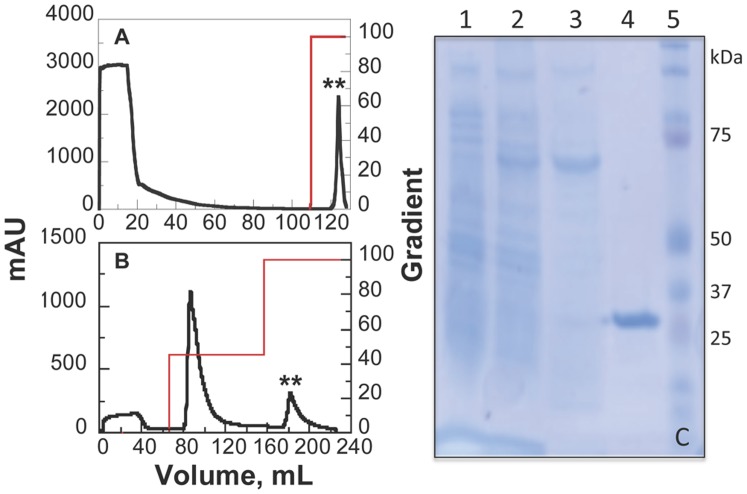
Purification of rGIFT15. Purification of His-MBP-GIFT15 on Nickel-Sepharose (A) and on DEAE-Sepharose (B) after cleavage with TEV protease. Black traces are the absorbance readings recorded at 280 nm and red traces represent the elution gradient. (C) SDS-PAGE following His-MBP-GIFT15 purification. (1) cell-free extract before induction with IPTG, (2) cell-free extract after induction with IPTG, (3) His-MBP-GIFT15 after Ni-Sepharose, (4) rGIFT15 after TEV protease treatment and isolation on DEAE-Sepharose, (5) molecular weight markers.

Mouse GIFT15 was isolated using nickel-chelate chromatography as the major purification step, followed by digestion with TEV protease and anion exchange chromatography on DEAE Sepharose. The cell-free extract was loaded onto a His-Trap affinity column to bind the poly-histidine tagged rGIFT15. To remove non-specific interactions between contaminating extract proteins and the matrix, the column was extensively washed with 10% buffer containing a high concentration of imidazole (buffer B). The recombinant protein was eluted with a gradient from 10 to 100% buffer B developed in 10 bed volumes. Recombinant His-MBP-GIFT15 was collected and analyzed by SDS-PAGE (Fig 3). The solution containing the pooled fractions was concentrated and diluted in 20 mM Tris-HCl, pH 8.0. The His-MBP-GIFT15 fusion was then treated for 48 h at 4°C with TEV protease to cleave the MBP carrier from the target protein. After the cleavage reaction, the digestion mixture containing the target protein was loaded onto DEAE-Sepharose for FPLC. The column was then washed with 10% buffer B (20 mM Tris-HCl, pH 8.0, 1 M NaCl) to remove the His-MBP and TEV protease and it was developed in a linear gradient from 10% to 100% buffer B in 10 bed volumes (Fig. 3). Recombinant mouse GIFT15 eluted at a buffer B saturation of approximately 20%. The purified mouse GIFT15 protein appears as a single band on an SDS-PAGE gel stained with Coomassie blue at ∼31-kDa (Fig. 3C). The purification process described above typically yielded high purity (>90%) protein samples with an average of 5 mg per liter of culture of endotoxin-free fusokine.

### Endotoxin removal and desalting

Protein solutions containing endotoxins were treated with 1% Triton X-114 on ice. The solutions were then warmed to 37°C, whereupon two phases formed. The Triton X-114 phase, containing the endotoxins, was separated by centrifugation at 3,000 g for 15 min. Three rounds of phase separation reduced the endotoxin content by 95–97% and less than 2.5 EU/mg could be detected using an LAL Chromogenic Endotoxin Quantitation Kit (Pierce). The small amount of detergent that remained in the protein solutions was removed by gel filtration on PD-10 columns equilibrated in phosphate buffered saline.

Alternatively, the application of 0.1% Triton X-114 in the washing steps of the affinity chromatography was successful at reducing endotoxins content and increased the recovery of the target protein.

**Figure 4:**
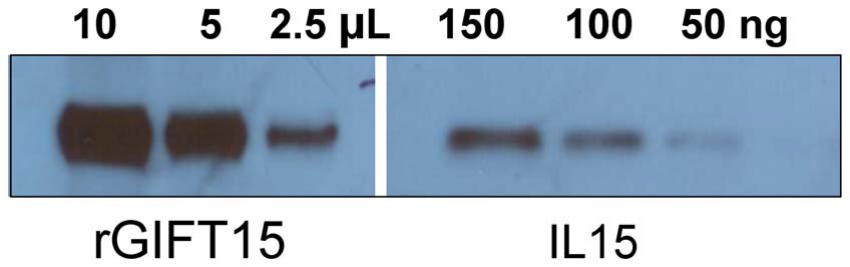
Western blot analysis of purified rGIFT15. Immunodetection was conducted with anti-mouse IL-15 antibody.

The final preparation was filter sterilized with a 0.22 µm syringe filter. Western blot analysis was performed on the final sample to quantify the amount of rGIFT15 by using recombinant mouse IL-15 or recombinant mouse GM-CSF as standards (Fig. 4).

### Biological activity of rGIFT15

**Figure 5:**
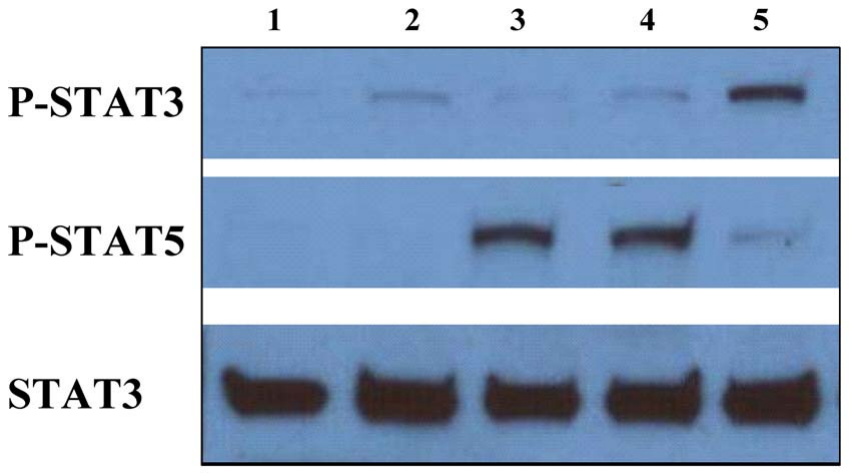
STAT3/5 phosphorylation in purified B cells. Purified B cells were stimulated for 1 h with (1) PBS or 30 pmol of (2) rIL-15, (3) rGM-CSF, (4) or both cytokines and (5) rGIFT15, and cell lysate was probed for the phosphorylation status of STAT3/5. Total STAT3 protein was used as a loading control.

To test the functional activity of rGIFT15, purified mouse splenic B cells were stimulated with the bacterially expressed, rGIFT15. On a molecular level, GIFT15 binds aberrantly to the trimeric IL-15 receptor (IL-15R), and as a result, asymmetrical signaling takes place downstream of the IL-15R, leading to hyperactivation of STAT3 and a hypo-STAT5 response [16]. Purified B cells collected from normal C57Bl/6 mice were utilized as GIFT15 responder cells and we investigated the phosphorylation status of STAT proteins following GIFT15 stimulation. Media containing 30 pmol cytokines was used to stimulate B cells for 1 h and cell lysates were used for Western blot analysis with antibodies specific to phosphorylated STAT proteins (Fig. 5).

### rGIFT15-induced Bregs

**Figure 6:**
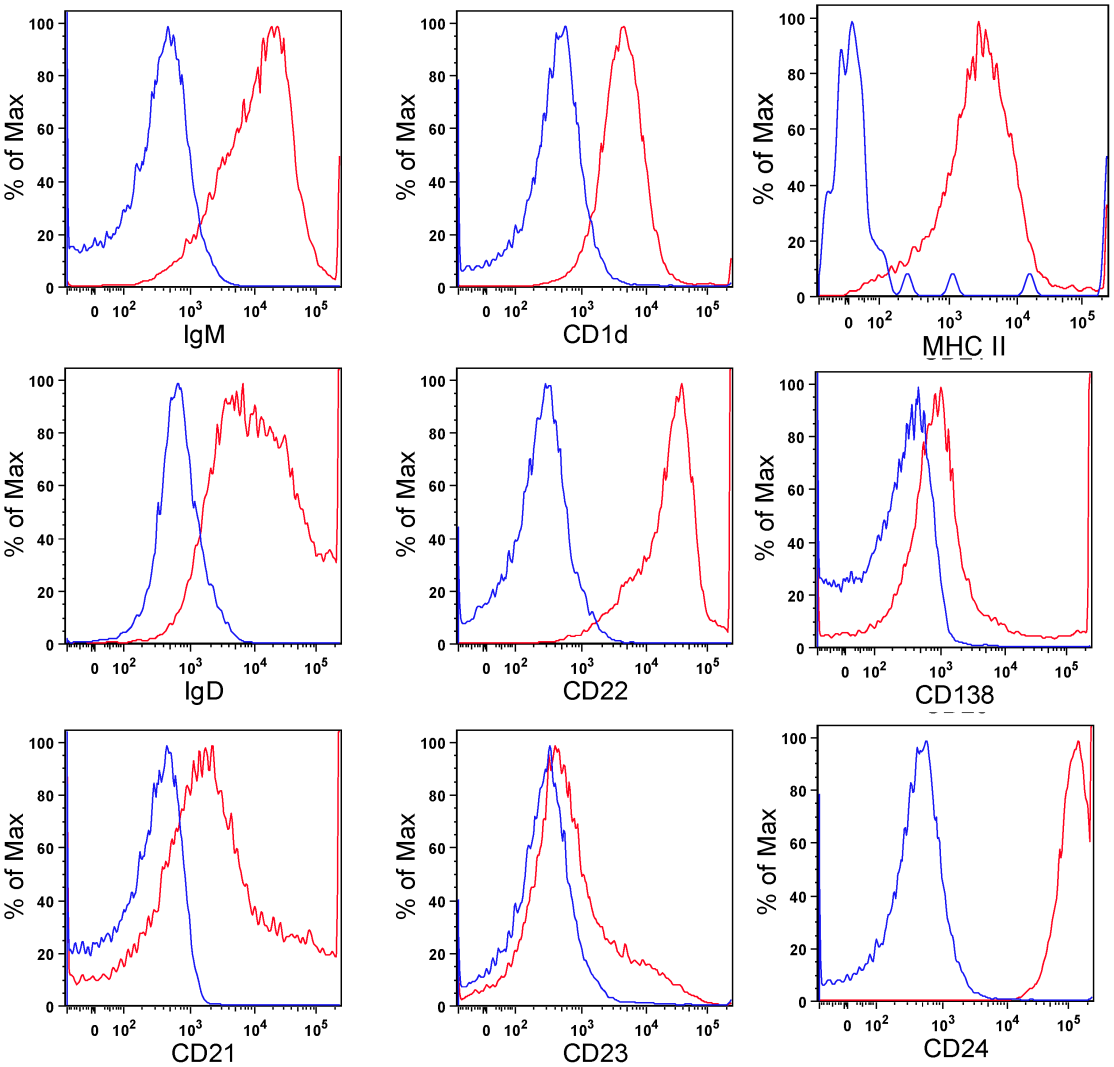
Flow cytometry analysis. FACS analysis of B cells purified from normal C57Bl/6 mice and then cultured for 5 d in the presence of rGIFT15. Blue line is the antibody isotype control; red line is the primary antibody.

GIFT15 possesses immunosuppressive properties and reprograms naïve B cells into regulatory B cells. Moreover, we demonstrated that rGIFT15-stimulated splenic B cells produce IL-10 *in vitro*. To identify the phenotype of the rGIFT15-treated lymphomyeloid cells, splenocytes collected from C57BL/6 (B6) mice were cultured for 5 d in the presence of GIFT15. To determine the phenotype of rGIFT15-treated B cells, we analyzed cells for the expression of surface B cell markers (Fig. 6). We found that rGIFT15-treated B cells were positive for Cd1d, CD21, CD22, CD23, CD24, CD138, IgM, IgD and MHC II marker proteins. The immune suppressive properties of Bregs arise from the secretion of soluble inhibitory factor IL-10 [30]. Therefore we used a mouse IL-10 ELISA to demonstrate the ability of GIFT15-derived Bregs to secrete IL-10 into the supernatant of the culture medium. As previously reported, significant amounts of IL-10 were detected when B cells were cultured with rGIFT15. The amount of IL-10 detected was 0.5±0.04 ng/mL in comparison to the negligible amount detected in cells treated only with PBS.

## Discussion

Biopharmaceutical development of protein agents has to consider many factors and risks. One of the most meaningful risks after formulation of the method to produce a pharmaceutical protein is the immunogenicity of the protein itself [31]. Among the several factors playing a role in immunogenicity, the presence of impurities, aggregates, and the degree of foreignness of the biopharmaceutical protein compared with the natural endogenous protein are all factors to be considered [32]. Whether the immune system considers the product self or non-self depends on differences in amino acid sequence, glycosylation and folding between the administered protein and its native homologue. A major concern associated with the use of refolded protein as therapeutic is that often refolding creates new epitopes compare to the native protein. All these differences can potentially be recognized as foreign by the recipient, and often leads to the induction of undesirable antidrug antibodies (ADAs) [33], [34].

Among different cytokines IL-15 and GM-CSF have been developed and tested in human clinical trials and GM-CSF is FDA-approved for clinical use [35], [36]. Both IL-15 and GM-CSF (as are most leukines and growth factors in clinical practice) were manufactured using a eukaryotic cell production system [1], [36]. Interferons are the only class of cytokines manufactured in bacterial fermentation systems that have obtained FDA marketing approval [37], [38]. These translational outcomes highlight the technical challenge of developing bacterial fermentation technologies for manufacture of bioactive leukine molecules, but also demonstrate the theoretical feasibility of bacterial expression systems to produce second generation engineered cytokines such as GIFT15. Since all the fusokines are chimeric proteins, the possibility that one domain may adversely interfere with the folding of the other one, led us to explore the possibility to generate soluble GIFT15 in *E. coli* bypassing the refolding process of inclusion body.

GIFT15 is a member of the GIFT fusokine family, which leads to the activation of lymphomyeloid cells with profound anti-inflammatory properties [15], [30]. To date, the mammalian cell systems used for the expression of GIFT15 allow for the secretion of the protein in unfractionated culture media. However, transfected mammalian cells are not a cost efficient system for producing GMP-compliant GIFT15 due to low expression levels and the presence of contaminating proteins secreted by the mammalian cells themselves. Considering the significant interest in developing non-traditional bioactive cytokines and protein derivatives, a cost-effective manufacturing approach is needed for pre-clinical and clinical development of promising lead compounds. In this study, we described an efficient method to produce large amounts of a bioactive recombinant GM-CSF and IL-15 chimeric polypeptide, GIFT15, in *E. coli*.

We tested different bacterial fermentation platforms in an attempt to produce bioactive GIFT15, taking into consideration production timescale, environmental containment, scalability, downstream processing strategy and overall costs [23], [39]. Heterologous expression in *E. coli* was selected as our first choice. Five constructs were tested in order to achieve high-level soluble expression of the recombinant protein. We observed that the GIFT15 fusokine was unstable in the *E. coli* cytoplasm. Only trace amounts were detectable by Western blot analysis when no tag was used or when a His-tag was added at the N-terminal of the protein. A likely cause of the observed instability may be an aberrant tertiary structure due to improper disulfide bridge structures within GIFT15. Indeed, disulfide bonds can be important stabilizing structures for proteins [23], [40], but the reducing environment of *E. coli* cytoplasm can be a challenge for the proper folding of recombinant proteins, leading to their accumulation within inclusion bodies [41]. Alternatively, misfolded proteins are quickly proteolyzed from the intracellular milieu. GIFT15 has 3 disulfide bonds, one of which is in the GM-CSF domain and the other two are in the IL-15 domain. To facilitate the formation of disulfide bounds, we fused GIFT15 with thioredoxin [42]. We found that the GIFT15-thioredoxin fusion was more abundantly expressed and protected GIFT15 from degradation. Unfortunately thioredoxin did not improve the solubility of the complete recombinant fusokine, as it also accumulated in inclusion bodies. Similar results were obtained when GIFT15 was fused with glutathione S-transferase.

The only strategy in which abundant expression of GIFT15 was observed as a soluble protein was the addition of MBP at the N-terminal of GIFT15. Our results are consistent with other studies in which MBP was found to be a strong enhancer of solubility when its expression is coupled with unstable proteins [29], [43]. Although the mechanism through which MBP increases the solubility of passenger proteins is still unclear, it is possible that MBP may prevent degradation by acting as a chaperone and allowing correct folding [29]. In the literature many human proteins have been successfully expressed in fusion with MBP and the purified proteins retain fully biological activity [42], [44]. Recently, large amounts of human GM-CSF were overexpressed and purified in fusion with the MBP in *E. coli* [45].

In our first attempts to purify the fusion protein, mouse GIFT15 was isolated with the use of a MBP-binding amylose column, followed by treatment with Factor Xa protease to cleave the MBP carrier. Anion exchange chromatography using DEAE-Sepharose was able to remove the recombinant GIFT15 from the protease. Unfortunately, most of the protein was lost in the flow-through of the amylose column [46]. Moreover, cleavage of the fusion protein by Factor Xa in other basic residues other than the consensus sequence Ile-Asp-Gly-Arg was observed [47], [48]. To address these issues, we kept MBP as a protein stabilizer and complemented it with the addition of a His-tag to allow for selectivity as part of a metal-chelate chromatography purification scheme. Substituting the Factor Xa cleavage site with that for the TEV protease allowed for efficient removal of tags without non-specific proteolytic degradation of the GIFT15 polypeptide sequence.

Functional bioassays on primary mouse splenic B-lymphocytes demonstrated that rGIFT15 exhibited biological activity comparable to mammalian-derived GIFT15. More specifically, we confirmed that stimulation of unfractionated mouse splenocytes or purified B cells with GIFT15 induced asymmetrical signaling on responsive lymphomyeloid cells downstream of the the IL-15 receptor, causing STAT3 hyperphosphorylation and STAT5 hypophosphorylation. Following tyrosine phosphorylation, STAT3 proteins dimerize, translocate to the nucleus, and activate specific target genes [49], [50]. Accordingly, STAT3 activation is associated with cell growth, anti-apoptosis, and pro-survival properties. Furthermore, GIFT15-stimulated Bregs expressed surface molecules [16] that are typical markers within a panel of inducible Bregs. Flow cytometry profiles of GIFT15-induced Bregs revealed their phenotype as CD1d, CD21, CD22, CD23, CD24, CD138, IgD, IgD and MHCII positive cells. These specific markers identify a subset of Bregs whose regulatory function is mediated by the production of IL-10 [16], [51] as we confirmed by detecting the release of IL-10 in the media of stimulated B cells.

Our findings describe a method that is permissive for the production of bioactive, highly purified, endotoxin-free rGIFT15 fusokine in a scalable bacterial process, making the procedure relevant for commercial, large-scale fermentation. Moreover, the addition of a MBP tag further expands the capability of *E. coli* to express different proteins such as complex fusokines as a source of valuable therapeutics. Our method of manufacture and purification clearly demonstrate that rGIFT15 deploys potent pharmaceutical properties akin to that observed with protein generated from eukaryotic cells.
